# Single-agent rituximab and ultra-low-dose adaptive radiotherapy for the treatment of indolent B-cell non-Hodgkin lymphomas

**DOI:** 10.3389/fonc.2025.1617087

**Published:** 2025-07-31

**Authors:** Katherine E. Lake, Meredith Jackson, Samantha Hull, Sean All, Akshat M. Patel, Xingzhe Li, Neil B. Desai, Elif Yilmaz, Heather Wolfe, Mohammad Faizan Zahid, Hsiao-Ching Li, Farrukh Awan, Margaret M. Kozak, Praveen Ramakrishnan Geethakumari, Kiran A. Kumar

**Affiliations:** ^1^ Department of Radiation Oncology, University of Texas Southwestern Medical Center, Dallas, TX, United States; ^2^ Department of Hematology and Oncology, University of Texas Southwestern Medical Center, Dallas, TX, United States

**Keywords:** iNHL, rituximab, radiotherapy, low-grade lymphoma, follicular lymphoma, ultra-low-dose, 4 Gy, boom boom

## Abstract

**Introduction:**

For indolent B-cell non-Hodgkin lymphomas (iNHLs), ultra-low-dose radiation therapy (ULDRT) with 4 Gy has demonstrated durable local control (70%), although distal relapses may occur. Concurrent systemic chemotherapy with radiation therapy (RT) extends progression-free survival (PFS) but is often avoided due to toxicity. We hypothesize that the combination of adaptive ULDRT, with repeat treatment as needed, and single-agent rituximab results in excellent local and systemic control with minimal toxicity.

**Methods:**

We conducted an institutional review board (IRB)-approved retrospective review of patients with iNHLs (n=26) who were treated with both ULDRT and rituximab (four weekly doses of 375 mg/m2), either concurrently or within a short interval (median 16 days), at our institution from 2017 to 2024. Treatment response and disease control (local and distant) were measured by PET/CT. Overall survival (OS) and PFS were analyzed using the Kaplan-Meier method. Common Terminology Criteria for Adverse Events (CTCAE) v4 was used to record acute and long-term toxicities.

**Results:**

Overall response rate (ORR) at the first follow-up was 28/31 (90%), of which 19 sites (61%) achieved complete response (CR) and nine (26%) achieved partial response (PR). One (3%) patient had stable disease (SD). In our cohort, the 2-year in-field, out-of-field, and overall PFS rates were 91%, 78%, and 78%, respectively, and OS was 92%. No patient had disease transformation.

**Discussion:**

The combination of rituximab and ULDRT demonstrates sustained local and distant disease control with minimal side effects in iNHLs.

## 1 Introduction

Indolent non-Hodgkin lymphomas (iNHLs) make up over 80% of all NHL cases (1). Focal radiation therapy (RT) is an effective treatment for local disease, although distal relapse is common (2). Alternatively, systemic therapy with chemotherapy provides local and distal control of disease but with the drawback of increased toxicity (3).

For the treatment of iNHLs, the FoRT trial demonstrated that 24-Gy RT has superior long-term local control when compared to ultra-low-dose radiation therapy (ULDRT). Sites treated with 24 Gy had higher response rates as well as longer time to progression. However, it is notable that local durable control was achieved in over 2/3 of sites treated with 4 Gy with significantly fewer toxicities and no difference in overall survival (OS) (4). Previous studies have shown that adding chemotherapy to RT significantly improved failure-free survival (5). The TROG study demonstrated that for stage I–II follicular lymphoma, the addition of systemic immunochemotherapy (rituximab, cyclophosphamide, vincristine, and prednisolone) following RT (30 Gy) decreased distal relapse and improved progression-free survival (PFS). However, systemic toxicities were common, especially those related to chemotherapeutic agents (3). Furthermore, prior retrospective review suggested that for stage I–II follicular lymphoma, the addition of four weekly doses of rituximab to RT (median 40 Gy) significantly increases PFS and is very well tolerated with minimal systemic toxicities (6).

The current National Comprehensive Cancer Network (NCCN) guidelines allow for many recommended treatment strategies for iNHLs (7). For example, for stage I or II follicular lymphoma, the NCCN recommends involved site radiation therapy (ISRT) alone (preferred), ISRT ± rituximab, or rituximab ± chemotherapy. Typical ISRT treatment strategies for follicular lymphoma consist of 24 Gy for “definitive” treatment; however, patients may experience acute and long-term RT-related toxicities with that dosage. For advanced-stage iNHLs, treatment strategies include active surveillance, palliative ISRT with 4 Gy, or rituximab ± chemotherapy. Recent studies have called for less toxic therapies, as treatment-related morbidity in patients with iNHLs can occur, and more aggressive treatment does not typically improve overall survival in these indolent lymphomas (8). At our institution, we have utilized ULDRT (4 Gy) in one or two fractions plus rituximab for iNHLs in select patients to provide both local and systemic control while limiting radiation-related toxicities, particularly those associated with treatment with 24 Gy.

For iNHLs, minimal data exist analyzing the use of a combination therapy of adaptive ULDRT with 4 Gy and single-agent rituximab, with retreatment as needed. The primary aim of this study was to evaluate the clinical outcomes in patients treated with this regimen, with the hypothesis that this regimen results in good local and systemic control with minimal toxicities.

## 2 Materials and methods

### 2.1 Participants and study design

This study was designed as an institutional review board (IRB)-approved retrospective review of patients with biopsy-proven iNHLs treated at our institution between February 2017 and February 2024. Patients were included if they underwent treatment with both ULDRT with 4 Gy in one or two fractions and rituximab (four weekly doses of 375 mg/m^2^) as frontline therapy, either concurrently or sequentially within a short period of time (median 16 days) as part of the same treatment strategy.

### 2.2 Procedures

Patient demographic, tumor, and treatment characteristics were collected. All patients were simulated for treatment using planning computed tomography (CT), and those with head and neck sites received appropriate immobilization. The radiation technique was chosen by the treating radiation oncologist. Patients were treated with either 3-dimensional conformal radiation (3D CR) therapy or intensity-modulated radiotherapy (IMRT). Treatment modality and energy were decided at the discretion of the treating radiation oncologist. All patients completed ULDRT (4 Gy, in one or two fractions) to the involved site and four weekly infusions of rituximab (375 mg/m^2^) as prescribed.

### 2.3 Outcomes

The primary outcome was both in- and out-of-field lymphoma control as measured by response assessment. Local control was defined as complete response (CR) or partial response (PR) by the Lugano classification criteria. The overall response rate (ORR) was those with CR or PR. Non-responders were defined as stable disease (SD) or progressive disease (PD). Secondary outcomes included OS, PFS, toxic effects, and symptom improvement.

Initial response was assessed at the first follow-up in an outpatient clinic. Local control and survival were continually assessed using radiographic imaging, including PET/CT, CT, and MRI as per standard-of-care guidelines. Patients were assessed for toxic effects at the time of treatment, as well as at each subsequent follow-up visit. Common Terminology Criteria for Adverse Events (CTCAE) v4.0 was used to grade acute and late toxicities.

### 2.4 Statistical analysis

For statistical analysis, each site was considered independent from the others. Descriptive statistics were used to summarize the baseline characteristics of the study cohort. OS and PFS were analyzed using the Kaplan–Meier method. Analysis was performed using Rv4.3.0.

## 3 Results

### 3.1 Clinical and treatment characteristics

There were 31 treatment sites from 26 patients with indolent NHL identified who met the inclusion criteria. Patients underwent treatment with both ULDRT (4 Gy in one or two fractions) and single-agent rituximab (4 doses) either concurrently or within a short interval (median 16 days). The median age was 72 years (range, 21–87) at the time of first treatment. The cohort was 65% male and 77% non-Hispanic White, with a median Eastern Cooperative Oncology Group (ECOG) score of 1 (range 0–2). Of the 26 patients, nine (35%) were stage I, eight (31%) were stage II, five (19%) were stage III, and four (15%) were stage IV. Lesions were classified by histological subtype as well as anatomic region. Of the 31 lesions, 15 (48%) were classified as follicular lymphoma, eight (26%) as mucosa-associated lymphoid tissue (MALT) lymphoma, and eight (26%) as nodal marginal zone lymphoma. Lesions were distributed across six major sites: 11 (35%) in the abdomen, seven (23%) in the head and neck excluding the parotid, five (16%) in the pelvis, four (13%) in the parotid, three (10%) in the chest, and one (3%) in the spine. Baseline clinical and treatment characteristics are summarized in **Table 1**.

**Table 1:** Baseline characteristics.

Disease characteristics		N (median (range)	%
Age (years)		72 (21–87)	
ECOG PS		1 (0–2)	
Gender	Male	17	65
	Female	9	35
Ethnicity	Hispanic	4	15
	Non-Hispanic, White	20	77
	Asian	1	4
	Black	1	4
Stage	I	9	35
	II	8	31
	III	5	19
	IV	4	15
Histologic subtype	Follicular	15	48
	Nodal MZL	8	26
	MALT lymphoma	8	26
Treatment sites	Pelvis	5	16
	Parotid	4	13
	Abdomen	11	35
	Chest	3	10
	Head and neck	7	23
	Spine	1	3

MZL, marginal zone lymphoma; MALT, mucosa-associated lymphoid tissue; PS, Performance Status.

Thirty-one lesions were treated with 4 Gy in one or two fractions. Treatment modalities consisted of 3D CR (n = 14, 45%) and IMRT (n = 17, 55%). Six patients had prior systemic therapy, including ibrutinib, pembrolizumab, R-CHOP (cyclophosphamide, doxorubicin, prednisone, rituximab, and vincristine), and bendamustine–rituximab. Seven patients had received RT for prior malignancies or prior involved sites. One patient received initial treatment of his/her iNHL with systemic rituximab therapy. As this patient experienced PD on this regimen, he/she was then given ULDRT, on which he/she achieved CR. Three patients continued maintenance rituximab therapy after completion of ULDRT. Two of these patients achieved CR on ULDRT and had pre-existing autoimmune diseases. The remaining patient achieved SD on initial RT treatment and elected for maintenance rituximab in place of salvage ULDRT due to worsening of pre-existing age-related functional and memory decline.

### 3.2 Response rates

Each treatment site was considered independent of the other for response assessment. The ORR at the first follow-up (median 2 months) was 28/31 (90%), of which 19 sites (61%) achieved CR and nine (29%) achieved PR. One (3%) patient had SD. Of those with PR, seven had residual disease in-field and two out-of-field. Three of these patients were re-treated with ULDRT, while two underwent systemic treatment with chemotherapy. Both progressions were out-of-field.

### 3.3 Survival

**Figure 1:**
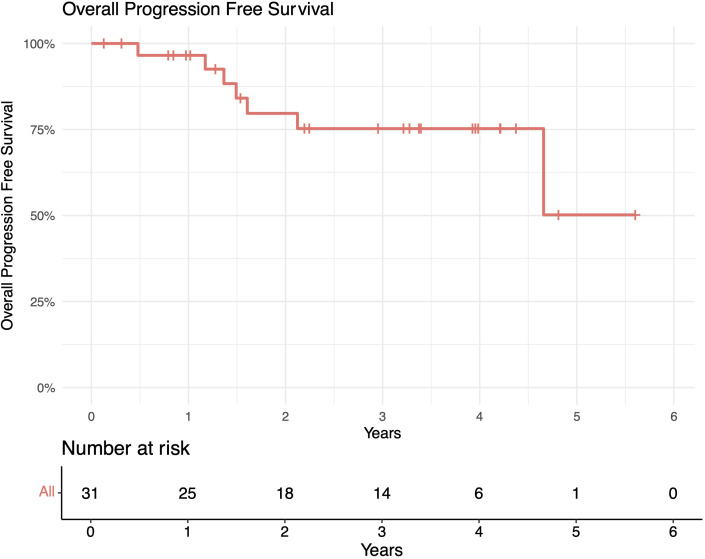
In-field progression-free survival (PFS) of patients with indolent B-cell non-Hodgkin lymphomas (iNHLs) after ultra-low-dose adaptive radiation therapy with 4 Gy and single-agent rituximab (four weekly doses of 375 mg/m^2^).

**Figure 2:**
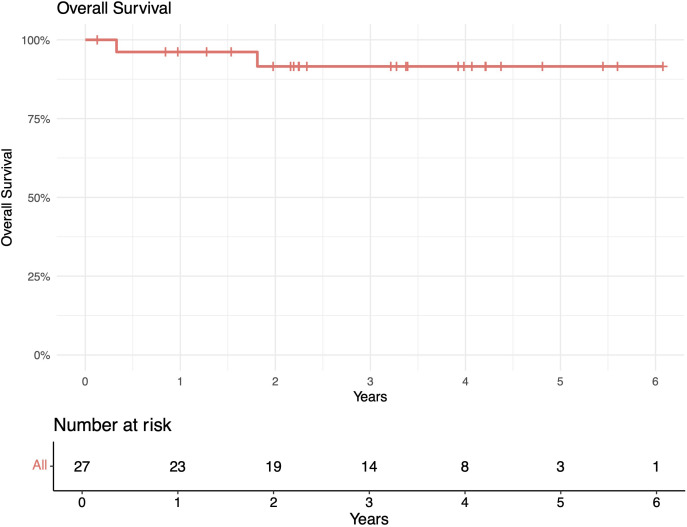
Out-of-field progression-free survival (PFS) of patients with indolent B-cell non-Hodgkin lymphomas (iNHLs) after ultra-low-dose adaptive radiation therapy with 4 Gy and single-agent rituximab (four weekly doses of 375 mg/m^2^).

**Figure 3:**
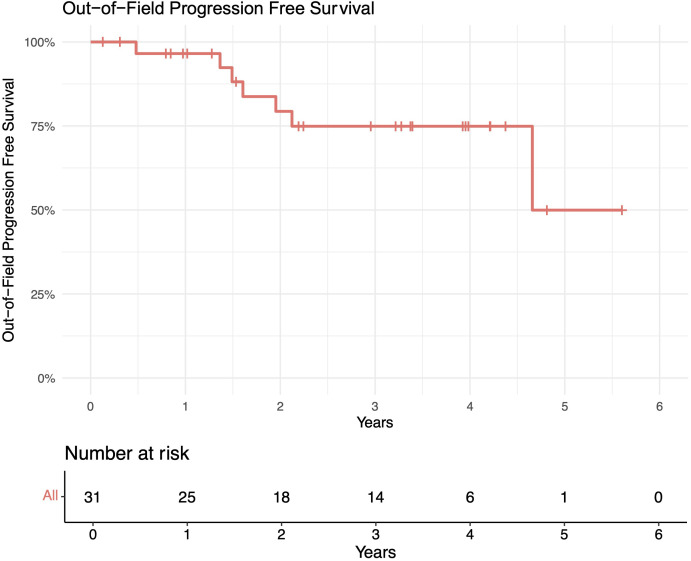
Overall progression-free survival (PFS) of patients with indolent B-cell non-Hodgkin lymphomas (iNHLs) after ultra-low-dose adaptive radiation therapy with 4 Gy and single-agent rituximab (four weekly doses of 375 mg/m^2^).

**Figure 4:**
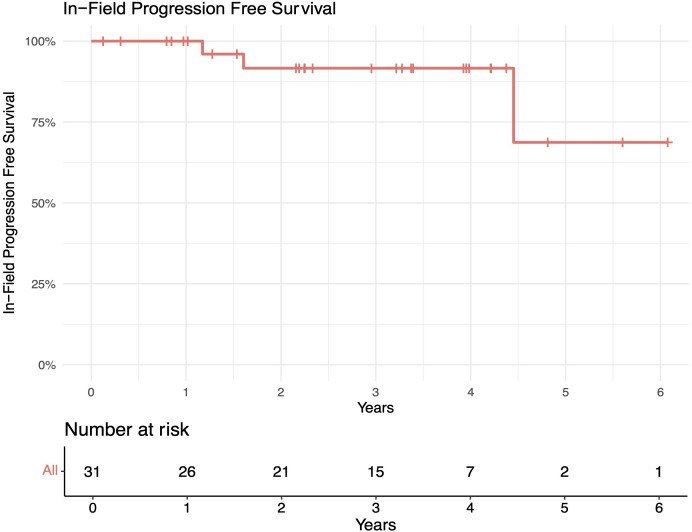
Overall survival (OS) of patients with indolent B-cell non-Hodgkin lymphomas (iNHLs) after ultra-low-dose adaptive radiation therapy with 4 Gy and single-agent rituximab (four weekly doses of 375 mg/m^2^).

The median follow-up length was 39 months (range 2–73). The 1-year in-field, out-of-field, and overall PFS rates were 100%, 96%, and 96%, respectively. The 2-year in-field, out-of-field, and overall PFS rates were 91%, 78%, and 78%, respectively (**Figures 1**–**4**). OS was 92%. The median time to any relapse was 1.5 years. Of the seven patients who relapsed, four relapsed out-of-field (57%), and three relapsed both in- and out-of-field of ULDRT (43%). Two of these patients were retreated with ULDRT, two underwent systemic treatment with bendamustine–rituximab, and one underwent retreatment with four weekly doses of rituximab. Of the five patients requiring retreatment, the average time to any treatment was 2.2 years (min: 163 days, max: 4.9 years), and the average time to systemic treatment was 3 years (min: 1.9 years, max: 4.9 years). No patient had disease transformation. Two patients died of complications unrelated to their iNHLs: one from complications of his/her known pre-existing myelodysplastic syndrome and the other from his/her known pre-existing Merkel cell carcinoma. No patient died of causes related to their iNHLs or treatment.

### 3.4 Toxicities and symptoms

Patients tolerated ULDRT well with no grade 3 or higher toxicities. Two patients (7%) experienced mild (grade 1–2) acute RT-related toxicities. One patient experienced diarrhea, which resolved within days. One patient experienced dysgeusia secondary to parotid RT, which resolved within 2 months. One patient (4%) noted chronic RT-related toxicity of mild chronic xerostomia, although this patient had pre-existing Sjögren’s syndrome. There were no major toxicities associated with systemic rituximab therapy.

Most treatment sites (20/31, 65%) were symptomatic prior to treatment. The median time to symptom improvement after completion of ULDRT was 2 months (range 0–5 months). Overall, 16/20 (80%) of lesions noted resolution of symptoms, one (5%) site noted improvement of symptoms, and three (15%) sites noted no change in symptoms. The site that demonstrated improvement without resolution was complicated by neurologic involvement of the iNHLs, and the patient had poor baseline functionality. The sites that did not demonstrate any improvement in symptoms were confounded by chronic degenerative changes, either present on imaging prior to iNHL diagnosis or caused by destructive metastases prior to treatment.

## 4 Discussion

In this study, we investigated the role of ULDRT in combination with rituximab for the treatment of iNHLs. Multiple other studies have assessed the response rates of iNHLs to chemotherapy, RT, or a combination of both therapies. One study’s results support the molecular rationale by which anti-CD20 immunotherapy with rituximab enhances RT and the combination synergizes to enhance cell growth delay and apoptosis in lymphoma (9). However, there are few studies evaluating the combination of RT and single-agent rituximab, of which most used higher doses of RT. The largest study available combining RT and single-agent rituximab is a multicenter observational study comparing patients with grade I and II follicular lymphoma treated with RT alone to patients treated with RT to an average of 40 Gy following treatment with rituximab (4 doses of 375 mg/m^2^). The results of the study showed that patients who underwent combination therapy had a significantly longer 10-year PFS. Our study is the largest to date reporting outcomes after local ULDRT with 4 Gy with concurrent single-agent rituximab.

Our response rate outcomes were comparable or superior to those of previously studied regimens (**Table 2**). Our initial response rates (CR 61% and CR plus PR 90%) were comparable to the results of the FoRT trial treatment with 24 Gy (CR 68% and CR plus PR 91%) and numerically superior to FoRT trial treatment with 4 Gy (CR 49% and CR plus PR 81%) (4). They were also numerically superior to the results of multiple studies examining the treatment of iNHLs with single-agent rituximab therapy, both at four weekly doses of 375 mg/m^2^, and with the addition of rituximab maintenance therapy, citing CR from 7% to 27% and overall response rates from 47% to 73% (10, 11). This suggests not only that combined therapy of RT and rituximab could have better initial response rates than ultra-low-dose RT alone but that higher levels of RT may not be needed to achieve initial site response when combined with rituximab. One similar study assessing eight doses of rituximab and involved field RT with 30 or 40 Gy had a superior response at the first follow-up (98.8%); however, 89% of patients in their study had acute RT side effects, compared to 9% of patients in this study (12).

**Table 2:** Initial response rates from this study, the FoRT trial, and two studies assessing the efficacy of rituximab alone (four doses of rituximab at 375 mg/m^2^).

Study	CR	ORR (CR + PR)
FoRT (24 Gy)	67%	91%
FoRT (4 Gy)	48%	80%
Hainsworth et al. (first-line rituximab)	7%	47%
Colombat et al. (first-line rituximab)	20%	73%
Lake et al. (4 Gy + rituximab)	61%	90%

Timing at initial response varied between studies (3 months, FoRT; 6 weeks, Hainsworth et al.; 50 days, Colombat et al.; and median 2 months, Lake et al).

CR, complete response; ORR, overall response rate; PR, partial response.

Our survival statistics (in-field 2-year PFS 91% and OS 92%) were comparable or improved compared to those of other studies (**Table 3**). The FoRT trial observed no difference in OS between patients who received 24 and 4 Gy, and patients who received treatment with 4 Gy had significantly fewer side effects, similar to our study (4). Additionally, they reported 2-year local progression-free rates (defined equally to in-field PFS) of 94% and 80% for 24 and 4 Gy, respectively. Our 2-year in-field PFS of 91% suggested durable local control with ULDRT plus rituximab. Additional studies should be performed to compare PFS and toxicity in iNHLs when treated with ULDRT plus rituximab vs. the standard of care with 24 Gy.

**Table 3:** Two-year in-field, out-of-field, and overall progression-free survival of this study and the FoRT trial.

Study	In-field 2-year PFS	Out-of-field 2-year PFS	Overall 2-year PFS
FoRT (24 Gy)	94%	—	—
FoRT (4 Gy)	80%	—	—
Lake et al. (4 Gy + rituximab)	85%	76%	76%

PFS, progression-free survival.

A prospective trial of patients with stage I–II iNHLs demonstrated that the combination of RT and chemotherapy increased 5-year OS (89% vs. 79%) and significantly increased PFS at 5 years (74% vs. 40%) when compared to patients who received RT alone (5). Prior to the FoRT trial, one study demonstrated that the median time to progression after treatment with ULDRT was 14 months, while our data demonstrated a median time to relapse (defined equally as the median time to progression) of 1.5 years (13). However, a study treating iNHLs with rituximab weekly for 4 weeks with 375 mg/m^2^ with the addition of maintenance rituximab as needed reported a PFS of 34 months (11). As a small subset of our patients later received maintenance rituximab, either due to inability to receive further salvage RT or for treatment of secondary comorbid conditions, the role of maintenance rituximab in place of salvage ULDRT is an area for further exploration.

Patients tolerated the combination of ULDRT and rituximab very well, with 2/26 (8%) experiencing acute toxicities and 1/26 (4%) chronic toxicity, although the one patient who experienced chronic toxicity had significant cofounding variables. Of note, while the incidence of acute low-grade (I–II) radiation-associated toxicities was comparable with that of other studies using ULDRT, we had no high-grade (III–IV) toxicities (4).

This study is limited by the relatively small sample size, retrospective design, limited follow-up time of 39 months, and potential variability in reporting treatment complications. Additionally, a small subset of patients continued to receive maintenance systemic therapy after the completion of our combination regimen. To account for this, the response assessment was analyzed at the first follow-up. Another limitation of this study is that the lesions are likely not independent of each other. While a cluster model could be used to account for this bias, we did not perform this analysis due to limited sample size and retrospective study design. Finally, our survival statistics are confounded by death due to pre-existing and terminal conditions as well as prior treatments. Prospective studies, such as the FoRT trial and others, excluded patients with limited predicted life expectancies and prior chemotherapies. As this was a retrospective study, we did not exclude deceased patients due to other causes or other treatments. Two patients in our study were deceased due to their pre-existing myelodysplastic syndrome and Merkel cell carcinoma. This likely decreased PFS and OS in our cohort compared to those of other studies due to increased patient frailty.

Prior studies have shown that the treatment of iNHLs with RT with 4 Gy using 2 Gy × 2 can achieve durable local disease control and that combining rituximab with RT in the treatment of patients with iNHLs increases PFS. However, prior studies combining RT and rituximab have used higher doses of therapeutic agents, which increases the risk for short- and long-term toxicities. In contrast, the use of ULDRT allows for local salvage treatment as needed, with decreased concern for long-term cumulative RT-related toxicities. To our knowledge, our study is the largest reported to date combining ULDRT and single-agent rituximab for the treatment of indolent lymphoma. Further studies are needed to assess the extent and clinical utility of synergy between ULDRT and single-agent rituximab. Additionally, prospective studies with larger sample sizes and longer follow-up are needed to define the optimal use of this treatment paradigm. The ongoing prospective GAZAI trial is one such effort evaluating adaptive involved-site radiotherapy with another anti-CD20 monoclonal antibody, obinutuzumab, in limited-stage follicular lymphoma (FL) (14). These studies should focus on exploring the possibility of specific subsets of patients with iNHLs who would benefit the most from its implementation.

In conclusion, our results show that in our study population, the combination of rituximab and ULDRT demonstrated sustained local and distant disease control over a median follow-up time of 39 months with minimal side effects in iNHLs. Therefore, in situations where there is concern for toxicity related to chemotherapy and/or higher radiation doses, this strategy could present a reasonable alternative to the standard first-line treatment of radiation therapy with 24 Gy.

## Data Availability

The datasets analyzed for this study can be found in the Zenodo repository, accession #14947948 (https://doi.org/10.5281/zenodo.14947948).
